# Microbiological Characteristics and Pathogenesis of *Klebsiella pneumoniae* Isolated from Hainan Black Goat

**DOI:** 10.3390/vetsci9090471

**Published:** 2022-08-31

**Authors:** Meirong He, Haoyang Li, Zhenxing Zhang, Junming Jiang, Hong Li, Weijie Yang, Yiwen Cheng, Hongyan Gao, Qiaoling Chen, Li Du, Si Chen, Churiga Man, Fengyang Wang

**Affiliations:** Hainan Key Laboratory of Tropical Animal Reproduction & Breeding and Epidemic Disease Research, Animal Genetic Engineering Key Lab of Haikou, College of Animal Science and Technology, Hainan University, Haikou 570228, China

**Keywords:** *Klebsiella pneumoniae*, microbiological characteristics, pathogenesis, bacterial load determination, RNA sequencing

## Abstract

**Simple Summary:**

*Klebsiella pneumoniae* (*K. pneumoniae*) can cause a multitude of infectious diseases in humans and animals. In this study, we isolated and identified a strain of *K. pneumoniae* from Hainan Black goat by using conventional microbiology techniques. Through these and combined with the RNA sequencing (RNA-seq) method, the molecular mechanism of the interaction between *K. pneumoniae* and animals was able to be better understood. The pathogenic mechanism of *K. pneumoniae* to animals was explored by establishing the mouse infection model. Our results revealed that *K. pneumoniae* induced bacteremia and mild pulmonary inflammation in mice by intraperitoneal injection. These results provided a theoretical foundation for subsequent diagnosis and treatment research on the diseases caused by *K. pneumoniae*.

**Abstract:**

*K. pneumoniae* is an opportunistic pathogen that leads to widespread infection in humans and animals, seriously threatening human health and animal husbandry development. In our research, we investigated the biological characteristics of the isolate by using a 16S rRNA gene sequencing, biochemical assay, and drug sensitivity test. Moreover, the pathogenicity study, including the bacteria load determination, the histopathology examination, and the RNA sequencing was carried out to explore whether the isolate could cause lung injury in mice through intraperitoneal injection. The results indicated that the isolate was identified as *K. pneumoniae* and named as KPHN001. The drug susceptibility test showed that KPHN001 was only sensitive to polymyxin B and furazolidone, and was resistant to other 28 antibiotics. In the bacteria load determination, the highest bacterial load of the organs was found in the spleen, and abundant bacterial colonization was also found in the lung. The histopathology showed the mainly acute inflammations in the lung were due to congestion, edema, and exudation. RNA-seq analysis revealed that the differentially expressed genes (DEGs) of inflammatory cytokines and chemokines were expressed massively in mice. In the present research, the biological characteristics and pathogenesis of clinically isolated *K. pneumoniae* were systematically studied, revealing the pathogenic mechanism of *K. pneumoniae* to animals, and providing a theoretical basis for the following prevention, control, and diagnosis research.

## 1. Introduction

*Klebsiella pneumoniae* (*K. pneumoniae*) is a gram-negative bacterium, and as an opportunistic pathogen, it is able to colonize in the respiratory tract or the intestinal tract of humans and animals [1]. It causes infectious diseases such as pneumonia, bacteremia, liver abscess, urinary tract infection, arthritis, and meningitis [2,3], posing a great threat to human health and livestock husbandry development [4]. At present, the two most prevalent pathogenic strains are classical *K. pneumoniae* (cKp) and hypervirulent *K. pneumoniae* (hvKp). Compared with hvKp, cKp is less virulent and usually damages immunocompromised patients [5], and it often evolves into multidrug-resistant *K. pneumoniae* (MDR-Kp) [6]. With the increasing prevalence of MDR strains, the clinical treatment of infectious diseases, such as pneumonia and bacteremia, has become increasingly difficult [7], which not only brings great obstacle to the clinical prevention and control of bacterial infection but also causes substantial economic losses to the livestock industry.

In order to improve the level of etiological diagnosis and treatment of *K. pneumoniae*, it has become a hot field of research to explore the damage caused by *K. pneumoniae* to animals under different routes of infection. *K. pneumoniae* is well known for its severe damage to the lung [8]. Moreover, as an initial infection, pneumonia generally leads to host bacteremia [9]. Among gram-negative bacteria, *K. pneumoniae* is often isolated from bacteremia cases secondary to pneumonia [10]. Therefore, to explore the infection of *K. pneumoniae* to the animal lung, the mouse model of pneumonia is often constructed by nasal drop, tracheal inoculation, or aerosol delivery [11,12,13], all of which are also common ways to construct a mouse pneumonic bacteremia model [10,14,15]. Nonetheless, *K. pneumoniae* is a pathogen that can cause host infection through a variety of infection routes. Therefore, whether respiratory tract infection is the only initial route to cause pneumonia in clinical practice remains to be further studied. In other studies, intraperitoneal and tail vein injections were used to simulate a liver abscess and bacteremia in mice [16,17], and yet, none of these studies showed whether intraperitoneal or tail vein injections could induce lung damage in mice.

Therefore, we isolated and identified a strain of *K. pneumoniae* from goat joint fluid by using conventional microbiology techniques in this study. To investigate the pathogenicity of the bacterium and whether an intraperitoneal injection leads to lung injury, a mouse model of *K. pneumoniae* infection was established. We tried to elucidate the infection mechanism from the perspectives of the visceral bacterial distribution and the changes in the gene expression profile of the mouse lung through the bacterial load determination and the RNA-seq method, so as to lay a reference for the subsequent prevention, control, diagnosis, and treatment.

## 2. Materials and Methods

### 2.1. Isolation and Identification of Bacteria

The samples were collected from the joint fluid of Hainan Black goats. The joint fluid samples were diluted in 500 μL phosphate buffer saline (PBS), and then 100 μL of diluent was spread on tryptic soy agar (TSA), blood agar, and MacConkey agar (Qingdao Haibo Biotechnology Co., Ltd., Qingdao, China), respectively. The morphology of colonies was observed after incubation at 37 °C for 18–24 h. Colonies were picked for 16S rRNA polymerase chain reaction (PCR) amplification. The sequences of bacterial 16S rRNA primer [18] were 27F: 5′-AGA GTT TGA TCC TGG CTC AG-3′, 1492R: 5′-GGT TAC CTT GTT ACG ACT T-3′. The length of the amplified product was 1466 bp. The PCR reaction system was 50 μL, including 25 μL Taq Master Mix, 2 μL upstream primer, 2 μL downstream primer, and 21 μL ddH_2_O. Single colonies were selected and mixed with the system, and then PCR amplification was performed. The PCR amplification procedure was as follows: 94 °C for 4 min, 94 °C for 30 s, 57 °C for 30 s, 72 °C for 90 s, 30 cycles, 72 °C for 10 min. After amplification, PCR products were electrophoresed on 1% (*w*/*v*) agarose gel. The products with matching length were sent to Haikou Nanshan Gene Biotechnology Co., Ltd. for sequencing. NCBI Blast (https://blast.ncbi.nlm.nih.gov/Blast.cgi, accessed on 4 August 2022) was used for sequence alignment to screen out the target strains. After 16S rRNA identification, single colonies were selected and inoculated in 5 mL tryptic soy broth (TSB) (Qingdao Haibo Biotechnology Co., Ltd., Qingdao, China) and incubated overnight at 37 °C with shaking at 180 revolutions per minute (rpm). Next, the bacterial solution was purified and cultured in TSA. After three generations of purification, single colonies were selected and fixed on a slide for Gram staining, and then the bacterial morphology was observed under a 100× oil microscope. Finally, the purified single colonies were selected and cultured in the corresponding biochemical microreaction tubes and incubated at 37 °C for 24–48 h to observe the color change in the reaction tubes.

### 2.2. Drug Sensitive Test

The susceptibility of the isolate to 30 antibiotics was detected by the Kirby–Bauer disk diffusion method according to the standards provided by the Clinical Laboratory Standards Institute (CLSI) Identification Manual (Version 2022).

### 2.3. Experimental Animals

Twenty 18–22 g healthy specific-pathogen-free (SPF) female Kunming mice were purchased from Hainan Institute of Medicine Co., Ltd. (number of animal license SYXK 2020-0025). Among them, 14 mice were used for pathogenicity studies and 6 mice were used for independent histopathology examination. In the pathogenicity test, 14 mice were allocated randomly into one challenge group (*n* = 7) and one control group (*n* = 7). In the histopathology examination, 6 mice were also divided randomly into one challenge group (*n* = 3) and one control group (*n* = 3). The mice were placed in ventilated, sterile cages and had free access to food and water. All experimental protocols were approved by the Academic Committee of Hainan University under the ethical approval code HNUAUCC-2021-00068.

### 2.4. Pathogenic Test of K. pneumoniae

The single colonies on TSA were selected and cultured in a 5 mL TSB medium. The bacteria was cultured overnight in the 180 rpm shaker at 37 °C. The optical density (OD) value of the bacterial solution was measured, and the viable amount of bacteria was cultured to 5 × 10^9^ colony-forming units (CFUs)/mL in combination with the results of plate colony count in the previous study. After enrichment by centrifugation (1743 reactive centrifugal force), the supernatant was discarded and the bacteria were suspended in PBS. Mice in the challenge group were injected with 0.2 mL of 1 × 10^9^ CFUs bacterial solution, while the ones in the control group were injected with 0.2 mL of PBS. The mental and death status of mice were observed and recorded after challenging.

### 2.5. Bacterial Colonization in Mice

To explore the distribution of visceral bacteria in mice challenged with *K. pneumoniae*, after the death of mice in the pathogenicity experiment, 3 mice in the challenge group and 3 mice in the control group were taken to collect the intact heart, liver, spleen, lung, and kidney, respectively. The connective tissue and fat were removed, and each organ was loaded into a 2 mL sterile Eppendorf (EP) tube filled with three 3 mm grinding beads, then weighed, and an appropriate amount of sterile PBS was added to make the mass of each tube the same. The EP tubes were put into the tissue lapping instrument and ground for 2 min. After grinding, PBS was replenished to 1 mL, and then the grinding solution was diluted to 10^−1^, 10^−2^, 10^−3^, 10^−4^, 10^−5^, 10^−6^, and 10^−7^, successively. Then tissue grinding solution with different dilutions of 100 μL was spread in TSA, and the results were observed and recorded after being cultured in a 37 °C incubator for 20 h.

In order to verify whether the strain separated from lungs was the strain used in challenge group, the colonies growing on TSA were selected for PCR identification of the specific gene *KHE* of *K. pneumoniae*. The primer sequences [19] were *KHE*-F: 5′-ATG AAA CGA CCT GAT TGC ATT CGC-3′, *KHE*-R: 5′-TTA CTT TTT CCG CGG CTT ACC GTC-3′. The length of the amplified product was 489 bp. PCR reaction system was the same as above. The reaction procedure was as follows: 95 °C for 3 min, 94 °C for 1 min, 55 °C for 45 s, 72 °C for 1 min, 30 cycles, 72 °C for 10 min.

### 2.6. Histopathology Examination of Lungs

To investigate whether intraperitoneal injection can cause pathological injury of mouse lung, 3 mice in the challenge group and 3 mice in the control group were selected for histopathology examination after the mice died. The lungs were aseptically collected, fixed in 4% (*v*/*v*) paraformaldehyde for 24 h, dehydrated in different concentrations of ethanol, and embedded in paraffin. The tissue was cut into 3 μm sections and then stained with hematoxylin and eosin (H&E). Finally, the sections were dehydrated and sealed for microscopic observation.

### 2.7. RNA Preparation and Sequencing

To explore the interaction between *K. pneumoniae* and the mouse immune system at the level of gene expression, intact lungs were collected from 3 challenge mice and 3 control mice after the mice in the challenge group died. Total RNA was extracted from mouse lung samples according to the instructions of Total RNA Extractor (Trizol) extraction kit (Sangon Biotech (Shanghai) Co., Ltd., Shanghai, China). The Qubit RNA Assay Kit (Life Technologies Corporation, Carlsbad, CA, USA) was used to detect RNA concentration, and 1% (*w*/*v*) agarose gel was used to detect RNA integrity and whether genomic was contaminated. The mRNA isolated from the complete total RNA of the sample was fragmented afterwards. Then the double-stranded cDNA was synthesized and purified, and the cDNA was modified by fragment and amplified into library. The amplified products of the cDNA library were detected by gel electrophoresis, and the recovered DNA was accurately quantified by the Qubit DNA Assay Kit (Life Technologies Corporation, USA). After testing and quality control, a sequencing library was obtained that was suitable for the Illumina platform.

### 2.8. RNA-seq Analysis and DEGs Identification

The quality of the original sequencing data was assessed by FastQC (V0.11.2), and the quality was trimmed by Trimmomatic [20] (V0.36) to obtain clean reads. The Q20, Q30, and GC contents of clean reads were calculated. HISAT2 [21] (V2.1.0) was used to compare the effective data of samples to the mouse reference genome (GRCm38), and the mapping information was counted. Sequences mapped to the genome were assembled using StringTie [22] (V1.3.3b) and then compared with the known gene models using GffCompare (V0.10.1) to construct intact transcripts. Gene expression was evaluated using StringTie and the known gene models. DESeq2 [23] (V1.12.4) was used for gene expression differential analysis, then the results of expression differential analysis were visualized. In order to obtain DEGs, the screening conditions were set to *p*-value < 0.05 and |log_2_FoldChange| > 1. The volcano map was drawn and cluster analysis was performed based on the results of the DEGs analysis. ClusterProfiler (V3.0.5) (Annoroad Gene Technology (Beijing) Co., Ltd., Beijing, China) was used for KEGG function enrichment analysis. When the *p*-value was <0.05, the function was considered to be significantly enriched. The top 20 pathways with the smallest *p*-value were selected, and the enriched distribution point map of KEGG pathway was drawn. GO enrichment of DEGs was performed by topGO alone (V2.24.0), and GO enrichment classification statistical map was drawn.

### 2.9. qRT-PCR Validation of DEGs

Total RNA was extracted from mouse lungs using the Total RNA Extractor (Trizol) extraction kit and reverse transcribed into cDNA. Twelve immune-related genes were selected from the DEGs (Table 1). Quantitative real-time PCR (qRT-PCR) was used to validate these DEGs, and *β-actin* was selected as the reference gene. Based on the reference sequences in NCBI, specific primers were designed using primer-blast and based on the reference sequences in NCBI (Table 1). The design criteria were: (a) the size of PCR product was from 80 to 200 bp; (b) melting temperature was 60 ± 2 °C; (c) primers must span exon–exon junctions. Equation F = 2^−(∆∆CT)^ was used to calculate the ratio of target genes to reference genes as the relative expression level of genes. Where ∆∆CT = experimental group (Ct _target gene_ − Ct _β__-actin_) − control group(Ct _target gene_ − Ct _β__-actin_).

### 2.10. Statistical Analysis

All data were represented as mean ± standard deviation (SD). GraphPadPrism8.0 was adopted for bacteria load determination (two-way ANOVA method) and qRT-PCR validation (multiple *t*-test). *p*-value < 0.05 was considered significant.

## 3. Result

### 3.1. Colonies and Morphological Characteristics of Isolated Bacteria

**Figure 1:**
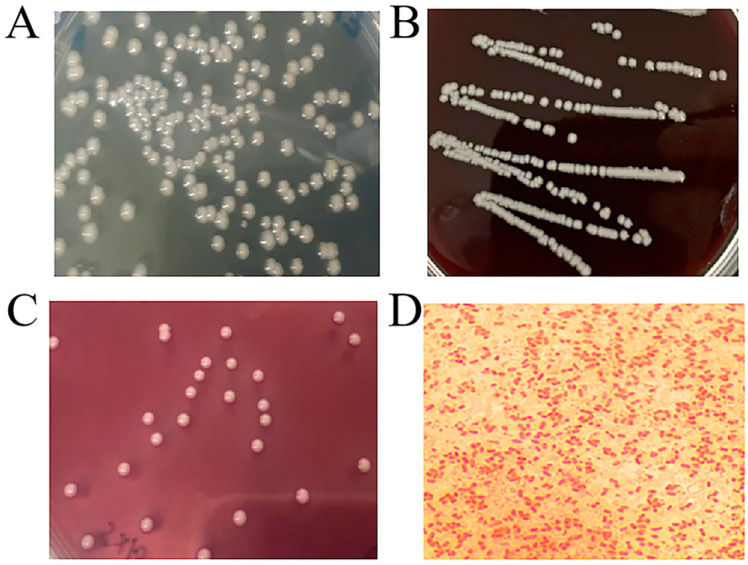
Culture colony morphology and Gram staining microscopic image of *K. pneumoniae*. (**A**) Colony morphology on TSA. (**B**) Colony morphology on blood agar. (**C**) Colony morphology on McConkey agar. (**D**) *K. pneumoniae* Gram staining microscopic morphology, 100×.

The isolated bacteria were cultured on TSA, blood agar, and McConkey agar for 24 h and grew well. Large, regular, round, smooth, raised, moist, and cream yellow colonies grew on TSA (Figure 1A). There was no hemolysis on the blood agar and the colonies were smooth, round, moist, ivory, and raised colonies (Figure 1B), while large, pink, regular, round, smooth, and raised colonies grew on McConkey agar (Figure 1C). The Gram staining result showed that the isolate was gram-negative bacillus. Single or short-chain permutation could be observed by microscopic examination (Figure 1D).

### 3.2. 16S rRNA Identification of Bacteria

**Figure 2:**
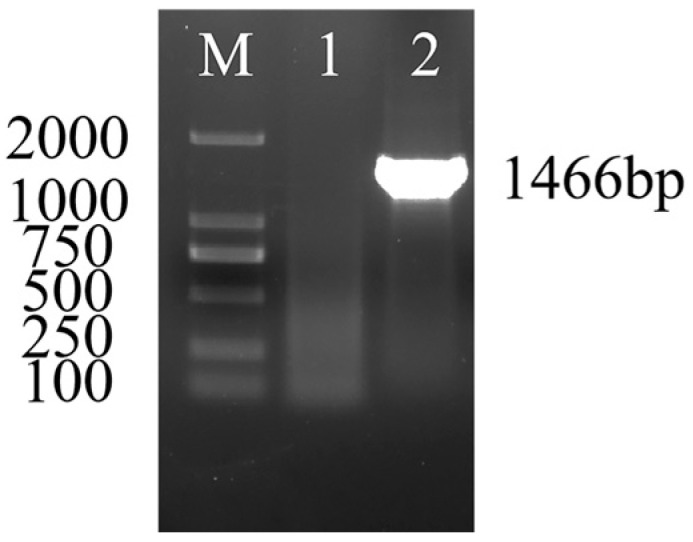
Results of 16S rRNA amplification electrophoresis of *K.pneumoniae*. “M” represents DL2000 DNA Marker. “1” represents negative control. “2” represents KPHN001 colony (The original gel figure can be found in Supplementary Figure S1).

The 16S rRNA gene of the isolate was amplified by PCR and the target band was 1466 bp in size by 1% (*w*/*v*) agarose gel electrophoresis (Figure 2), which was consistent with the size of the intended target fragment. Then the PCR products were sequenced. The alignment of the sequencing result was performed by Blast on NCBI and the results showed that the isolates had more than 99% homology with F16KP0070, A16KP0016, C17KP0055, and other *K. pneumoniae* reference strains in the database. The isolated bacteria could be preliminarily identified as *K. pneumoniae* and named as KPHN001.

### 3.3. Biochemical Identification of Bacteria

The single colonies were purified and cultured in biochemical microreaction tubes. The results could be judged after culturing for 24–48 h (Table 2). The results showed that KPHN001 was able to decompose some kinds of carbohydrate such as glucose, sucrose, maltose, etc. The indole test was negative, the methyl red test (MR) was negative, while the Voges–Proskauer (V-P) test was positive. All these results were consistent with the biochemical characteristics of *K. pneumoniae*.

### 3.4. Drug Sensitive Test of KPHN001

According to the standard for judging the diameter of the bacteriostatic ring in the drug susceptibility test provided by CLSI (Supplementary Table S1), the sensitivity of KPHN001 to the following 30 antibiotics was determined. The results revealed that KPHN001 was merely sensitive to polymyxin B and furazolidone, while it showed different degrees of drug resistance to penicillin, cefoperazone, ofloxacin, gentamicin, kanamycin, and 28 other drugs (Table 3).

### 3.5. Mouse Pathogenicity Test

**Figure 3:**
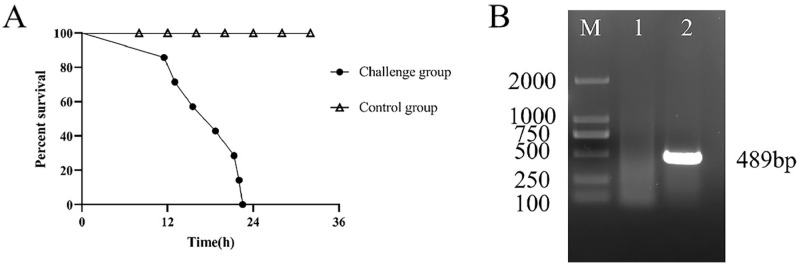
Pathogenicity test results of *K.* *pneumoniae*. (**A**) Line chart of survival rate of *K.* *pneumoniae*-infected mice. (**B**) Electrophoresis results of *KHE* gene amplification in *K.* *pneumoniae*. “M” represents DL2000 DNA Marker. “1” represents negative control; “2” represents TSA single colony (The original gel figure can be found in Supplementary Figure S1).

All the mice in the challenge group showed clinical symptoms, including purulent eye secretions, diarrhea, fecal paste sticking to the anus, crouching for warmth, and an obviously decreased appetite after challenging for 8–12 h. All the mice (*n* = 7) died in 12–24 h after being challenged (Figure 3A). In the control group, no abnormalities were found in mice and no death was observed (*n* = 7). The visceral tissue grinding solution was spread on the TSA medium and it was cultured for 20 h at 37 °C. Homogeneous single colonies could be seen on the medium. The single colonies of lung bacteria were picked for *KHE* specific gene PCR verification of *K. pneumoniae*. A specific band of 489 bp appeared in agarose gel electrophoresis (Figure 3B), indicating that this isolated bacteria was consistent with the bacteria for challenge that had a strong, lethal effect on mice.

### 3.6. Bacteria Load in Viscera of Mice

**Figure 4:**
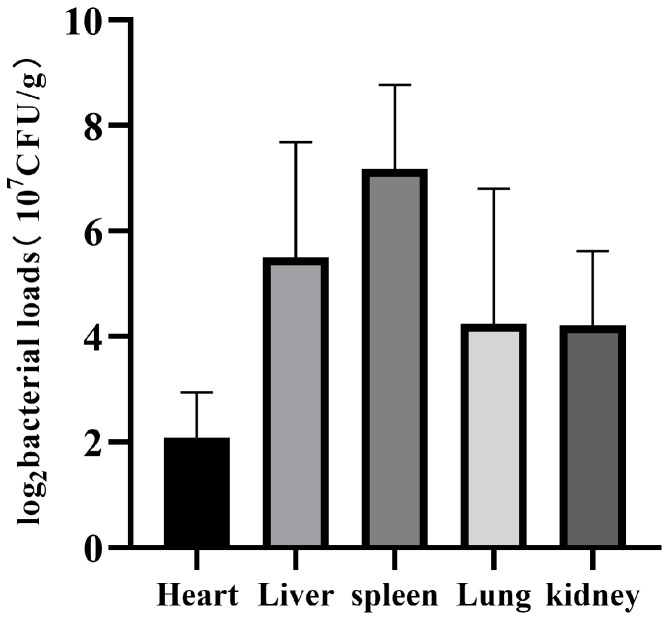
Average bacteria load in the five tissues of three mice.

The results of the bacteria load are shown in Table 4 and Figure 4. The hearts, livers, spleens, lungs, and kidneys of mice were colonized by a large number of bacteria in the challenge group after mice died, while in the control group, no bacteria were colonized in the mouse viscera. In the challenge group, the highest average bacteria load was found in the spleen, which was 2.21 × 10^9^ CFU/g. The bacteria load in the liver ranked the second highest, followed by the lung and kidney, and the heart had the lowest bacteria load.

### 3.7. Histopathology Examination of Mouse Lungs

**Figure 5:**
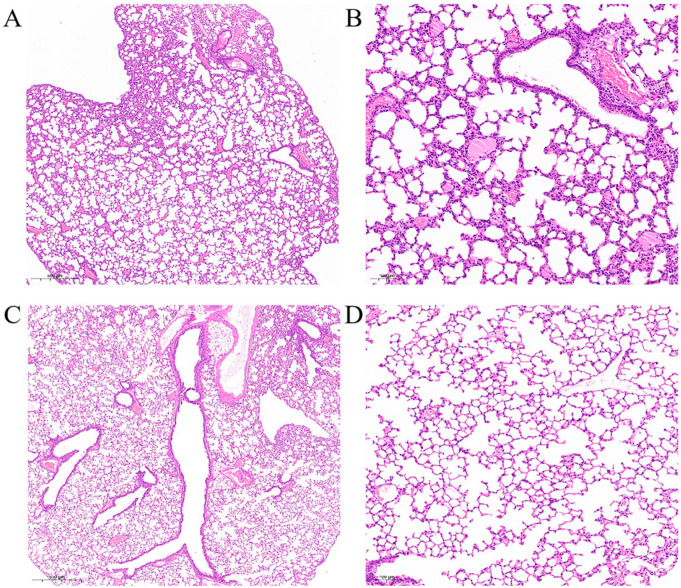
Mouse lungs histopathology with H&E staining. (**A**) Figure of lungs in challenge group, 3.0×; (**B**) figure of lungs in challenge group, 10.0×. The cellulose exuded in a few alveolar, vascular congestion, and perivascular inflammatory edema occurred in challenge group lungs. (**C**) Figure of lungs in control group, 3.0×; (**D**) figure of lungs in control group, 10.0×. Compared with challenge group, no obvious lesion could be examined in the lungs.

H&E staining results of mouse lung histopathologic slides showed that, compared with the control group, cellulose exuded in a few alveolar, vascular congestion, and perivascular inflammatory edema occurred after challenge with *K. pneumoniae* (Figure 5).

### 3.8. RNA-seq Data Analysis

The Total RNA of six mouse lungs (challenge group: three, control group: three) was extracted and used for the construction of a cDNA library. The library was sequenced using the Illumina high-throughput next-generation sequencing platform. After the removal of low quality reads and all possible contamination, clean reads were more than 55,000,000, Q30 > 96.16%, and GC percentage was between 51.11% and 51.72% (Table 5). The data from this study have been deposited to the NCBI Sequence Read Archive database with accession number PRJNA863221.

**Figure 6:**
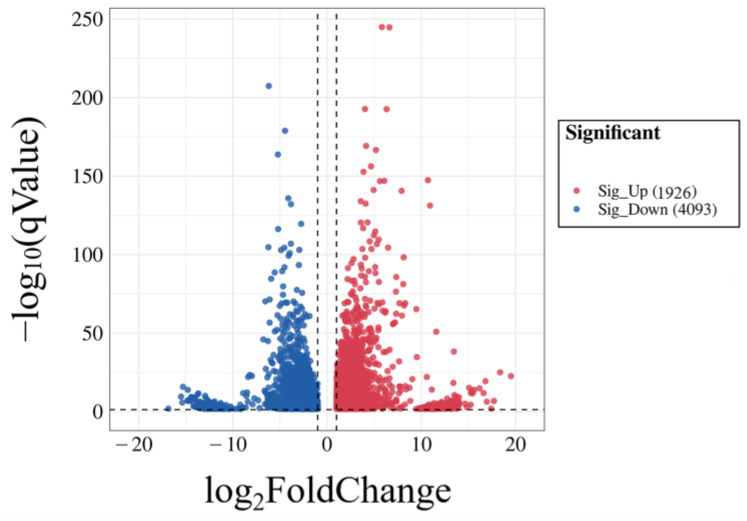
Volcano diagram of comparison group expression difference. Each dot in the figure represents a gene. Red dots represent up-regulated genes, while blue dots represent down-regulated genes.

In order to obtain differentially expressed genes (DEGs), we set the screening conditions as: *p*-value < 0.05 and |log_2_FoldChange| > 1. The result showed that in the challenge group, 1926 genes were up-regulated, while 4093 genes were down-regulated compared with the control group (Figure 6).

### 3.9. Gene Ontology and Kyoto Encyclopedia of Genes and Genomes Analysis

**Figure 7:**
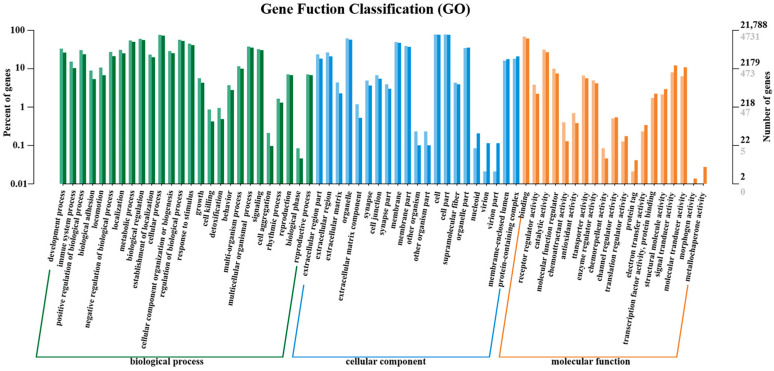
Histogram of GO annotation classification of differential genes. The horizontal axis is functional classification, and the vertical axis is the number of genes in the classification (**right**) and their percentage in the total number of genes annotated (**left**). Different colors represent different categories. On the bars and axes, the light colors represent DEGs and the dark colors represent all genes.

A total of 6019 DEGs were detected, and these DEGs were enriched into GO and KEGG, respectively. GO enrichment analysis was performed on the screened DEGs (Figure 7). Three parts of the GO database described the biological process, the cellular component, and the molecular function. Among them, “cellular process” was the most significantly enriched ontology in the biological process, with 3590 genes enriched. A total of 3692 genes were enriched in the “cell”, which was the most significantly enriched pathway in the cellular component, while the most significantly enriched ontology in molecular function was “binding”, with 3238 genes enriched in total (Figure 7).

**Figure 8:**
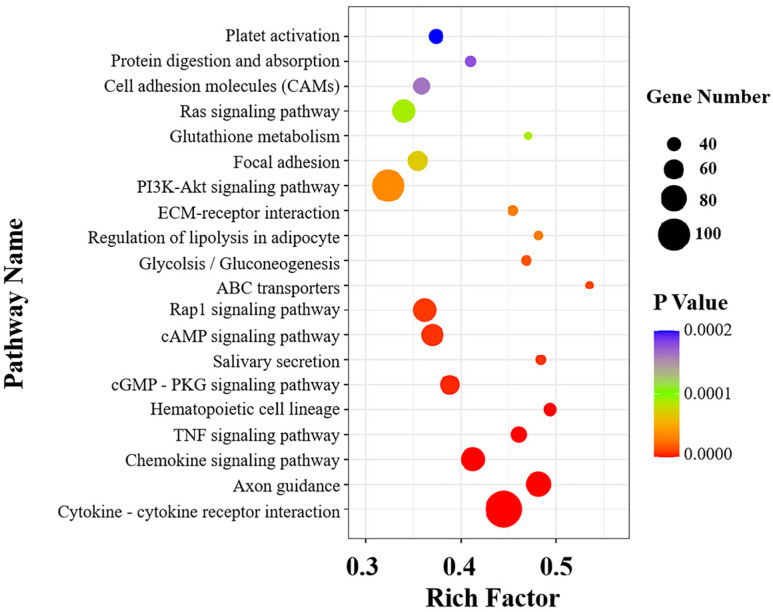
Functional scatter diagram of significant enrichment of KEGG. The vertical axis represents the annotation information of the function, and the horizontal axis represents the rich factor corresponding to the function (the number of different genes annotated to the function was divided by the number of genes annotated to the function). The size of *p*-value is represented by the color of the point. The smaller the *p*-value is, the closer the color is to red. The number of different genes contained in each function is indicated by the size of the dots.

KEGG enrichment results showed that 1499 DEGs were enriched in the KEGG pathway. According to the *p*-values from small to large, the top 20 KEGG pathways with the most significant differences were selected to draw Figure 8. Among the top 20 KEGG pathways, 10 pathways were immune-related. The most significantly differential pathway was the cytokine and cytokine receptor interaction with 113 DEGs, followed by the chemokine signaling pathway and the TNF signaling pathway, with 73 DEGs and 47 DEGs, respectively.

### 3.10. qRT-PCR Validation of DEGs

**Figure 9:**
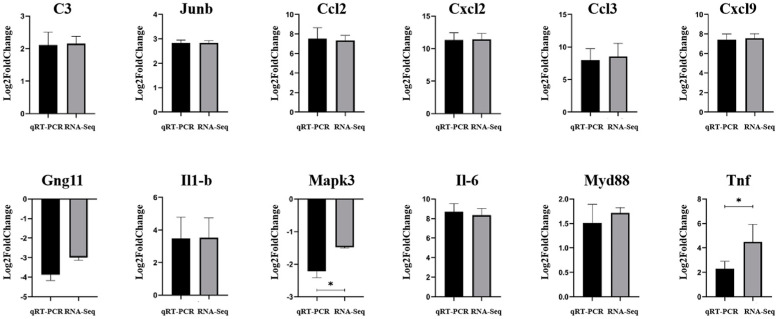
qRT-PCR results of the target gene. Black represents qRT-PCR verification results, gray represents RNA-seq results. Up-facing pillars indicate the gene expression was up-regulated, while the down-facing ones indicate the gene expression was down-regulated. * represents *p* < 0.05.

Twelve genes related to immunity were selected in order to evaluate the accuracy and reliability of the RNA-seq results. The results of qRT-PCR validation showed that the mRNA expression of *C3*, *Junb*, *Ccl2*, *Cxcl2*, *Ccl3*, *Cxcl9*, *Il1β*, *Il6*, *Myd88*, and *Tnf* were up-regulated, while the mRNA expression of *Gng11* and *Mapk3* were down-regulated. All these results were consistent with the RNA-seq data, which confirmed the reliability of the RNA-seq data in this study (Figure 9).

## 4. Discussion

*K. pneumoniae* is one of the most common gram-negative pathogenic bacteria that can cause humans and animals to become infected with various diseases [2,3]. In particular, when the host’s immunity is reduced or the long-term addiction to a large number of antibiotics leads to dysbiosis of bacteria, it is more likely to cause host infection, under which circumstances if the treatment is improper, it could lead to death [6]. In this study, 16S rRNA gene sequencing and biochemical test were used to identify the isolated bacteria. A strain of *K. pneumoniae*, named KPHN001, was isolated from the joint fluid of the Hainan Black goat in tropical regions for the first time. The drug sensitivity test of KPHN001 showed that KPHN001 was only sensitive to polymyxin B and furazolidone, and showed different degrees of resistance to the other 28 antibiotics, suggesting that KPHN001 was a multidrug-resistant strain.

In the present research, a mouse infection model was established by intraperitoneal injection in order to explore the pathogenic mechanism of KPHN001. Although different routes of infection affected the distribution of bacteria in the viscera to a great extent, Vornhagen et al. [14] and Anderson et al. [17] reported that whether the infection was via tail vein injection or intraperitoneal injection, the dynamics of infection in mouse both showed that *K. pneumoniae* could replicate extensively in the mouse spleen and liver. Moreover, the bacteria load in the spleen increased rapidly after infection, and showed a trend of significant increase over time [17]. It is worth noting that the blood, the liver, and the spleen have different microenvironments. For example, the metabolic flexibility of *K. pneumoniae* was mainly transmitted through the citrate synthase gene *GltA*, which was a key mediator necessary for *K. pneumoniae* to colonize in the liver and spleen, and yet this gene was dispensable in the blood [14]. However, no matter how the mice were infected, once the bacteria colonized the tissues and organs extensively, it meant that the mice were infected with bacteremia. In the *Streptococcus* *pneumoniae* infection mouse model established by Carreno et al. [15], it was found that bacterial replication in CD169^+^ macrophages in the spleen triggered persistent bacteremia. In our study, bacterial load was determined using the organs collected right after the mice died. The result revealed that the organs of three mice with the highest average bacterial load were the spleen (2.21 × 10^9^ CFU/g), followed by the liver (9.48 × 10^8^ CFU/g) and the lung (4.54 × 10^8^ CFU/g). The kidney (2.53 × 10^8^ CFU/g) and the heart (4.72 × 10^7^ CFU/g) were also largely colonized by bacteria. Moreover, as an important blood filter organ, the spleen was of great research value. In the present research, after large-scale replication in the mouse spleen, *K. pneumoniae* was eventually distributed in various tissues and organs of the body through blood circulation, suggesting that the mouse body had a high probability of bacteremia infection. Simultaneously, combined with the H&E staining results of lung histopathologic slides, it was suggested that the inflammatory reaction of the lung was mainly acute inflammation, such as congestion, edema, and exudation. However, the overall inflammation was mild, indicating that the intraperitoneal injection route had definite limitations in lung infection with *K. pneumoniae*.

In order to further explore the bacteria–host immune system interaction mechanism involved in the *K. pneumoniae* infection mouse model, we elucidated its possible mechanism from the perspective of a gene expression profile by RNA-seq technology. In our research, RNA-seq was applied to mouse lungs infected with KPHN001. The results display that, compared with the control group, substantial changes were observed in the transcriptome of mouse lungs in the challenge group and a total of 6019 DEGs were detected; among them, 1926 DEGs were up-regulated, while 4093 DEGs were down-regulated (*p*-value < 0.05). The results also indicated that half of the top 20 most significantly differential KEGG pathways were immune-related, including the cytokine and cytokine receptor interaction, the chemokine signaling pathway, and the TNF signaling pathway, indicating that these pathways play an important role in the inflammatory process of *K. pneumoniae*.

Quantitative analysis of twelve DEGs in these important pathways showed that *Ccl2*, *Ccl3*, *Cxcl2*, *Cxcl9*, *Tnf*, *Il1β*, *Il6*, and *Junb* were significantly up-regulated. These cytokines had a wide range of biological activities that contribute to coordinate the body’s response to infection. Among them, chemokine, as a chemotactic cytokine, could recruit inflammatory cells from the inside of blood vessels through endothelial and epithelial cells into the inflammatory site [24]. Chemokines such as CCL2 and CCL3 have been proven to regulate monocyte/macrophage and neutrophil recruitment in a variety of inflammatory diseases [25]. Chua et al. [26] conducted single-cell RNA sequencing of respiratory tract samples from severe coronavirus disease 2019 (COVID-19) patients and found that the expression of chemokines and their receptors in different cell populations significantly increased, including CCL2, CCL3, CCL20, CXCL1, CXCL3, and CXCL10. These results indicate that chemokines play a crucial part in the inflammatory response. Moreover, as an important proinflammatory cytokine, TNF-α is a key factor in the pathophysiology of cytokine release syndrome (CRS, also known as cytokine storm) [27]. Makwana et al. [28] found that the inhalation of TNF-α in healthy guinea pigs triggered an increase in neutrophil recruitment, consequently causing airway hyperresponsiveness and respiratory inflammation. TNF-α is also able to promote the production of cytokines such as IL-1β and IL-6 [29]. As one of the most important pleiotropic proinflammatory cytokines, IL-1β plays an important role in inflammatory diseases [27]. The effects of IL-1β are very similar to those of TNF-α, which could promote the production of various hematopoietic factors, especially IL-6 [30]. Additionally, IL-1β is capable of enhancing the expression of chemokines and adhesion molecules in mesenchymal cells and endothelial cells, sequentially recruiting immunocompetent cells to infiltrate into injured tissues [31]. IL-6, another pleiotropic cytokine, could stimulate the growth and differentiation of B lymphocytes and promote platelet production, which also activates the hepatocytes and induces the secretion of inflammatory proteins, such as reactive protein C (CRP) and fibrinogen [32]. Thus, IL-6 is involved in the regulation of the immune system, hematopoiesis, and inflammation, and has another pivotal role in CRS [27]. In addition to the above-mentioned proinflammatory cytokines, the AP-1 transcription factor JunB has attracted much attention for its important role in several biological processes, such as placenta formation and bone homeostasis [33]. Since JunB could be phosphorylated and activated by JNK in immune cells [34], research from Thomsen et al. [35] suggested that JunB was a relevant downstream JNK target in immune cells, regulating IFN-γ expression during acute hepatitis. In summary, these important chemokines and proinflammatory cytokines were widely involved in various biological processes and exerted complex cascade effects in response. In this study, the up-regulated genes during *K. pneumoniae* infection were associated with an immune inflammatory response, suggesting that *K. pneumoniae* infection could induce the overexpression of proinflammatory cytokines and chemokines, consequently causing cytokine storm.

To sum up, the pathogenic mechanism of *K. pneumoniae* was intimately related to the route of infection. In this study, the *K. pneumoniae* infection mouse model was successfully established by intraperitoneal injection. However, the limitation of this approach was that the bacteria could not reach the target organ, the lung, rapidly, which meant the mice died too soon to show obvious pathological damage. In our research, we thought that *K. pneumoniae* infection in mice caused bacteremia resulting in systemic multiorgan acute failure. Combined with RNA-seq data, it was suggested that a complex immune response was produced in vivo. The significantly enriched immune pathways and high expression of a large number of inflammatory cytokines were related to the cytokine storm caused by bacteremia.

## 5. Conclusions

In this study, we successfully isolated and identified a multidrug-resistant *K. pneumoniae* strain from goat articular fluid. The pathogenic mechanism of this strain on the animal was further studied by a mouse pathogenicity test, viscera bacterial load determination, and RNA-seq. The analysis of DEGs in *K. pneumoniae* infection helped us understand the molecular mechanism of the interaction between *K. pneumoniae* and animals. These results provided a unique insight into the mechanism of *K. pneumoniae* infection and laid a theoretical foundation for subsequent diagnosis and treatment research. However, these identified candidate pathways involved in the mechanism of function against *K. pneumoniae*, still require further investigation.

## Figures and Tables

**Table 1:** Information of genes and corresponding primers.

Name of Genes	Primer Sequence (5′-3′)	Product Length (bp)
*Cxcl2*	F: CACTCTCAAGGGCGGTCAA	96
	R: AGTTAGCCTTGCCTTTGTTCAG	
*Ccl3*	F: TCCCAGCCAGGTGTCATTTTC	105
	R: GGCATTCAGTTCCAGGTCAGT	
*Il1β*	F: AATGCCACCTTTTGACAGTGATG	139
	R: AGCTTCTCCACAGCCACAAT	
*Il6*	F: TGATGGATGCTACCAAACTGGA	199
	R: CTGTGACTCCAGCTTATCTCTTG	
*C3*	F: ACTTCTTCATTGACCTGCGGC	197
	R: CGAGGACTTGGGAGGGATTT	
*Tnf*	F: CCCTCACACTCACAAACCAC	134
	R: ACAAGGTACAACCCATCGGC	
*Ccl2*	F: CACTCACCTGCTGCTACTCA	117
	R: GCTTGGTGACAAAAACTACAGC	
*Cxcl9*	F: GTGTGGAGTTCGAGGAACCCT	173
	R: GGCAGGTTTGATCTCCGTTC	
*Myd88*	F: AAGCAGCAGAACCAGGAGTC	150
	R: GCAGTAGCAGATAAAGGCATCG	
*Junb*	F: CAGCCTTTCTATCACGACGAC	96
	R: GGTGGGTTTCAGGAGTTTGTAG	
*Gng11*	F: CAAGTTGCAGAGACAACAGGTATC	130
	R: GCTGCCCTTTTCTTTGAAGGG	
*Mapk3*	F: CAACACCACCTGCGACCTTA	154
	R: GGATTTGGTGTAGCCCTTGGAA	
*β-actin*	F: CCTCTATGCCAACACAGT	146
	R: TAGGAGCCAGAGCAGTAA	

**Table 2:** Results of KPHN001 biochemical test.

Biochemical Project	Result	Biochemical Project	Result
Glucose	+	Sorbitol	+
Sucrose	+	Urea	+
Maltose	+	Malonate	+
Mannose	+	Citrate	+
Arabinose	+	Ornithine	−
Raffinose	+	Lysine	+
Rhamnose	+	Indole	−
Xylose	+	Methyl red test (MR)	−
Mannitol	+	Voges–Proskauer test	+
Salicin	+	Hydrogen sulfide	−
Inositol	+	Nitrate reduction	+

“+” represents positive, while “−” represents negative.

**Table 3:** Results of KPHN001 drug sensitivity test.

Antimicrobial Agents	Inhibitory Zone Diameter/mm	Drug Resistance	Antimicrobial Agents	Inhibitory Zone Diameter/mm	Drug Resistance
Penicillin	0	R	Ciprofloxacin	14	R
Oxacillin	0	R	Vancomycin	0	R
Ampicillinum	0	R	Polymyxin B	17	S
Carbenicillin	0	R	Trimethoprim	0	R
Piperacillin	10	R	Furazolidone	19	S
Cefalexin	0	R	Chloramphenicol	0	R
Cefazolin	0	R	Amikacin	0	R
Cefradine	0	R	Gentamicin	0	R
Cefuroxime	0	R	Kanamycin	0	R
Ceftazidime	15	R	Neomycin	0	R
Ceftriaxone	9	R	Tetracycline	7	R
Cefoperazone	12	R	Doxycycline	0	R
Midecamycin	0	R	Minocycline	10	R
Norfloxacin	10	R	Erythromycin	0	R
Ofloxacin	12	R	Clindamycin	0	R

“S” represents sensitivity, while “R” represents resistance.

**Table 4:** Bacterial load in the five tissues of three mice.

Serial Number	Heart Bacteria Load (CFU/g)	Liver Bacteria Load (CFU/g)	Spleen Bacteria Load (CFU/g)	Lung Bacteria Load (CFU/g)	Kidney Bacteria Load (CFU/g)
E1	7.34 × 10^7^	3.07 × 10^8^	6.16 × 10^8^	1.79 × 10^8^	1.59 × 10^8^
E2	2.25 × 10^7^	1.27 × 10^8^	9.88 × 10^8^	3.27 × 10^7^	7.60 × 10^7^
E3	4.58 × 10^7^	2.41 × 10^9^	5.04 × 10^9^	1.15 × 10^9^	5.25 × 10^8^

**Table 5:** Sequencing data statistical summary.

Serial Number	Total Reads	Q10 Bases Ratio(%)	Q20 Bases Ratio(%)	Q30 Bases Ratio(%)	GC Bases Ratio(%)	N Bases Ratio(%)
C1	71,187,134	100.00%	98.98%	96.12%	51.62%	0.00%
C2	55,851,916	100.00%	99.01%	96.21%	51.69%	0.00%
C3	55,792,564	100.00%	98.99%	96.17%	51.72%	0.00%
E1	63,352,576	100.00%	99.03%	96.27%	51.38%	0.00%
E2	61,343,784	100.00%	98.98%	96.1%	51.69%	0.00%
E3	58,422,366	100.00%	98.98%	96.1%	51.11%	0.00%

## Data Availability

All data are presented in the article, and the original data can be obtained by email asking the author.

## Supplementary Materials

The following supporting information can be downloaded at: https://www.mdpi.com/article/10.3390/vetsci9090471/s1, Figure S1: Original gel figure, Table S1: Standard for judging the diameter of bacteriostatic ring in the drug susceptibility test by disk method.
