# Supplementary material for: Microbiological Characteristics and Pathogenesis of Klebsiella pneumoniae Isolated from Hainan Black Goat
Source: Vet Sci. 2022 Aug 31;9(9):471. doi: 10.3390/vetsci9090471 (PMC9501091; doi:10.3390/vetsci9090471)
Supplement: Supplementary file 1 [file vetsci-09-00471-s001.zip › vetsci-1876489-supplementary.pdf]

Figure S1. Original gel figure.

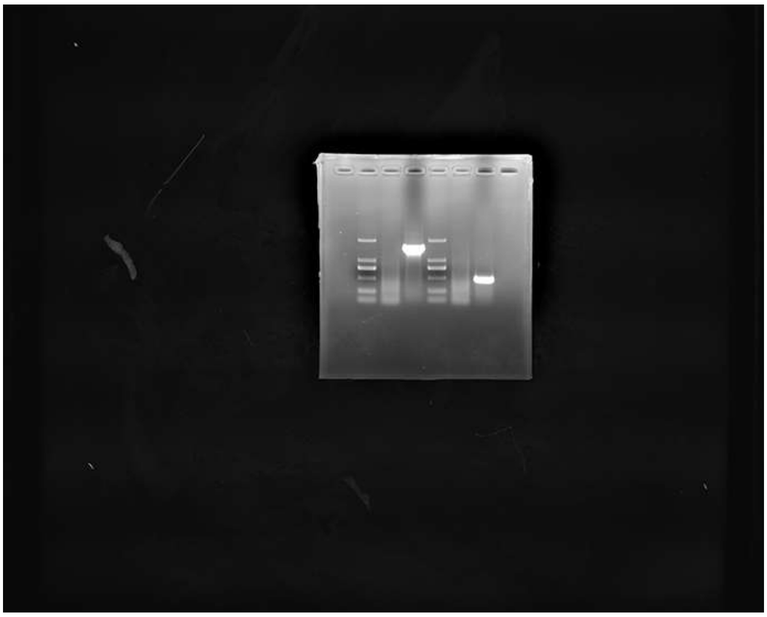

Table S1. Standard for judging the diameter of bacteriostatic ring in the drug susceptibility test by disk method.

Standard for judging the diameter of bacteriostatic ring in the drug susceptibility test by disk method.				
**Name of antibiotic**	**Paper content (ug/piece）**	**Zone Diameter Breakpoints (mm)**		
		R	I	S
Penicillin	10	≤14	-	≥15
Oxacillin	1	≤17	-	≥18
Ampicillinum	10	≤13	14-16	≥17
Carbenicillin	100	≤19	20-22	≥23
Piperacillin	100	≤17	18-20	≥21
Cefalexin	30	≤14	15-17	≥18
Cefazolin	30	≤19	20-22	≥23
Cefradine	30	≤14	15-17	≥18
Cefuroxime	30	≤14	15-17	≥18
Ceftazidime	30	≤17	18-20	≥21
Ceftriaxone	30	≤19	20-22	≥23
Cefoperazone	75	≤15	16-20	≥21
Midecamycin	30	≤13	14-17	≥18
Norfloxacin	10	≤12	13-16	≥17
Ofloxacin	5	≤12	13-15	≥16
Ciprofloxacin	5	≤21	22-25	≥26
Vancomycin	30	≤14	15-16	≥17
Polymyxin B	300	≤8	9-11	≥12
Trimethoprim	5	≤10	11-15	≥16
Furazolidone	300	≤14	15-16	≥17
Chloramphenicol	30	≤12	13-17	≥18
Amikacin	30	≤14	15-16	≥17
Gentamicin	10	≤12	13-14	≥15
Kanamycin	30	≤13	14-17	≥18
Neomycin	30	≤17	18-22	≥23
Tetracycline	30	≤11	12-14	≥15
Doxycycline	30	≤10	11-13	≥14
Minocycline	30	≤12	13-15	≥16
Erythromycin	15	≤13	14-22	≥23
Clindamycin	2	≤14	15-20	≥21
